# The genetic basis of water‐use efficiency and yield in lettuce

**DOI:** 10.1186/s12870-021-02987-7

**Published:** 2021-05-27

**Authors:** Annabelle Damerum, Hazel K. Smith, GJJ Clarkson, Maria José Truco, Richard W. Michelmore, Gail Taylor

**Affiliations:** 1 Department of Plant Sciences, University of California, Davis, 95616 CA USA; 2 School of Biological Sciences, University of Southampton, Hampshire, SO17 1BJ UK; 3 Present address: Vitacress Salads, Lower Link Farm, St Mary Bourne, SP11 6DB Hampshire, UK; 4 The Genome Centre, University of California, Davis, 95616 CA USA

**Keywords:** *Lactuca sativa*, Water‐use efficiency, Quantitative trait loci, Carbon isotope discrimination, Crop breeding, Leafy vegetable, Salad, Sustainable agriculture

## Abstract

**Background:**

Water supply limits agricultural productivity of many crops including lettuce. Identifying cultivars within crop species that can maintain productivity with reduced water supply is a significant challenge, but central to developing resilient crops for future water-limited climates. We investigated traits known to be related to water-use efficiency (WUE) and yield in lettuce, a globally important leafy salad crop, in a recombinant inbred line (RIL) lettuce mapping population, produced from a cross between the cultivated *Lactuca sativa* L. cv. Salinas and its wild progenitor *L. serriola* L.

**Results:**

Wild and cultivated lettuce differed in their WUE and we observed transgressive segregation in yield and water-use traits in the RILs. Quantitative trait loci (QTL) analysis identified genomic regions controlling these traits under well-watered and droughted conditions. QTL were detected for carbon isotope discrimination, transpiration, stomatal conductance, leaf temperature and yield, controlling 4–23 % of the phenotypic variation. A QTL hotspot was identified on chromosome 8 that controlled carbon isotope discrimination, stomatal conductance and yield under drought. Several promising candidate genes in this region were associated with WUE, including aquaporins, late embryogenesis abundant proteins, an abscisic acid-responsive element binding protein and glutathione S-transferases involved in redox homeostasis following drought stress were also identified.

**Conclusions:**

For the first time, we have characterised the genetic basis of WUE of lettuce, a commercially important and water demanding crop. We have identified promising candidate genomic regions determining WUE and yield under well-watered and water-limiting conditions, providing important pre-breeding data for future lettuce selection and breeding where water productivity will be a key target.

**Supplementary Information:**

The online version contains supplementary material available at 10.1186/s12870-021-02987-7.

## Background

Adapting to increases in the frequency and severity of drought stress, and the predicted rise in the cost and restricted availability of irrigation water, will require plant breeding that focuses on traits for drought tolerance and improved water-use efficiency (WUE). Water supply limits crop production worldwide more than any other environmental factor and has thus been the focus of many breeding efforts, but these have often been impeded, because identifying the ideotype for droughted or reduced water supply environments is complex. Timing of reduced water supply, duration and severity of drought all impact on crop ideotype [1]. Much of the mechanistic research on model plants such as Arabidopsis, although of value in elucidating candidate genes, often provides limited insight into the complex and highly crop-specific ideotypes required for breeding. The problem is complicated by the explicit link between water-use and yield, especially under water-limited conditions, such that breeding for restricted water supply can result in a significant yield penalty [2, 3].

Since lettuce cultivation began almost 5,000 years ago, the crop has spread globally with much diversification for different phenotypes through traditional selective breeding, both intra- and inter-specifically [4], paralleled by a gradual loss of genetic diversity in cultivated lettuce [4]. Within the genus, *Lactuca*, *L. serriola* L. has the greatest sexual compatibility with *L. sativa* and is the probable wild progenitor [5]. These species are fully interfertile, yet they have distinct phenotypes. *L. sativa* is a leafy plant with round leaves, no spines and a low latex content, whilst the weedy *L. serriola* is spiny and has a high bitter latex content [6]. Here, we have employed a recombinant inbred line (RIL) mapping population, which was produced using *L. sativa* and *L. serriola* as parents, as previously described by Truco et al. [7]. The parents have been shown to also differ in their root phenotypes; *L. sativa* has a shallow root system with much lateral branching, while *L. serriola* has a long taproot and is considered drought tolerant [8–10]. Wild species of lettuce have already been the source of disease resistance genes in breeding programs [11]. The natural genetic variation which exists in wild relatives has been explored previously in lettuce to identify genes regulating root architecture [12], seed and seedling traits [13–15], shelf life and processability [16], disease resistance [17] leaf and seed morphology [6, 18] and nutritional quality [19] and these have been incorporated into breeding programs [20]. Yet, wild species of lettuce have not been characterised for water-use traits, apart from those associated with root architecture [12].

The genetic control of water-use traits has been investigated in wheat, rice, maize, barley, tobacco, *Arabidopsis*, grape, soybean, sorghum, canola and cotton, with some progress made in elucidating the physiological relationship between yield and water limitation and identification of quantitative trait loci (QTL) for WUE traits [21–25]. Cultivars with enhanced production in water-limited environments have also been identified [26–28], but progress overall has been slow in deploying new fit-for-purpose varieties. Little research has focused on lettuce water-use or in understanding the mechanisms of drought tolerance in lettuce [6, 12]. The research that has been completed has tended to target head characteristics (for whole head lettuce), rather than baby leaf crops, which now represent a rapidly expanding market, in contrast to whole head iceberg lettuce which is declining in the United States [29–31].

Stomatal activity is a fundamental process which controls the interaction of a plant with its environment with respect to temperature and water regulation [32]. As water deficits increase, stomatal aperture declines, leading to an increase in leaf temperature [33]. It is this associated change in leaf thermal energy fluxes which can be monitored through thermal imaging [33] acting as a sensitive indicator of response to stress. Given that stomatal responses can occur prior to any change in plant water status, it is proposed to be a sensitive, pre-symptomatic indicator of soil water deficits [32]. Stomatal conductance (g_s_) has been found to be negatively correlated with leaf infra-red thermography measurements for a variety of crops, providing strong evidence that this proximal measurement can be a powerful indicator of the control of leaf water loss [33–38]. Although in the long-term, such data may be acquired remotely from satellites, currently these have a low pass rate, rely on clear days, require atmospheric correction and also have a large pixel size, meaning that the data is not necessarily applicable to the relatively small fields common in salad production. Field-based proximal imaging provides a high-throughput, non-destructive alternative, which does not impact upon g_s_. This paper compares leaf temperature measurements made using porometry and thermal imaging (infra-red, IR) in order to investigate the potential of field-based imaging as a viable tool for irrigation management and genetic screening in baby leaf salad systems.

Alongside water-use-related traits such as transpiration (E), leaf temperature and yield, both instantaneous and whole plant WUE may provide insight into drought tolerance. Since stomatal closure initiated by drought stress leads to a decrease in CO_2_ uptake, plants with good WUE are preferable in water scarce environments. Maximising WUE alongside yield is the primary focus of developing water productive plants, which could tolerate drought stress whilst still accumulating sufficient biomass to make their production commercially viable. Plant WUE can be estimated by comparing the ratio of the two stable isotopes of carbon, ^12^ and ^13^ C, since plants inherently discriminate against ^13^ C, with a lower ratio of ^13^ to ^12^ C observed in plant biomass compared to the atmosphere [39]. When intercellular CO_2_ decreases due to stomatal closure, higher ratios of ^13^ C are utilised, resulting in reduced carbon isotope discrimination (Δ^13^C). Plants with a lower Δ^13^C exploit more carbon per unit of water transpired, demonstrating an increased WUE [39]. Determining water use efficiency using Δ^13^C is an attractive option as the trait is highly heritable and an integrative measurement which can be taken at a single point in time [40]. Here, we aim to determine the genetic basis of Δ^13^C in lettuce and its relationship to yield in this economically important species, with the goal of improving the sustainability of water-use in baby leaf cropping systems.

A rich database of genetic and genomic resources are now available for lettuce, including a reference quality genome, along with a RIL mapping population developed from a cross between wild and cultivated lettuce (*L. sativa* cv. Salinas x *L. serriola* US96UC23), which has been exploited in several independent QTL mapping studies worldwide [6, 16, 17, 19, 41]. Utilising these resources in a quantitative genetics approach, this study is the first pre-breeding step to produce a more water-use efficient crop which can maintain growth under drought stress, with the potential to improve irrigation management and at the same time, provide candidate genes for future breeding for more sustainable lettuce germplasm.

## Results

### Contrasting water‐use patterns identified in wild and cultivated lettuce

**Fig. 1:**
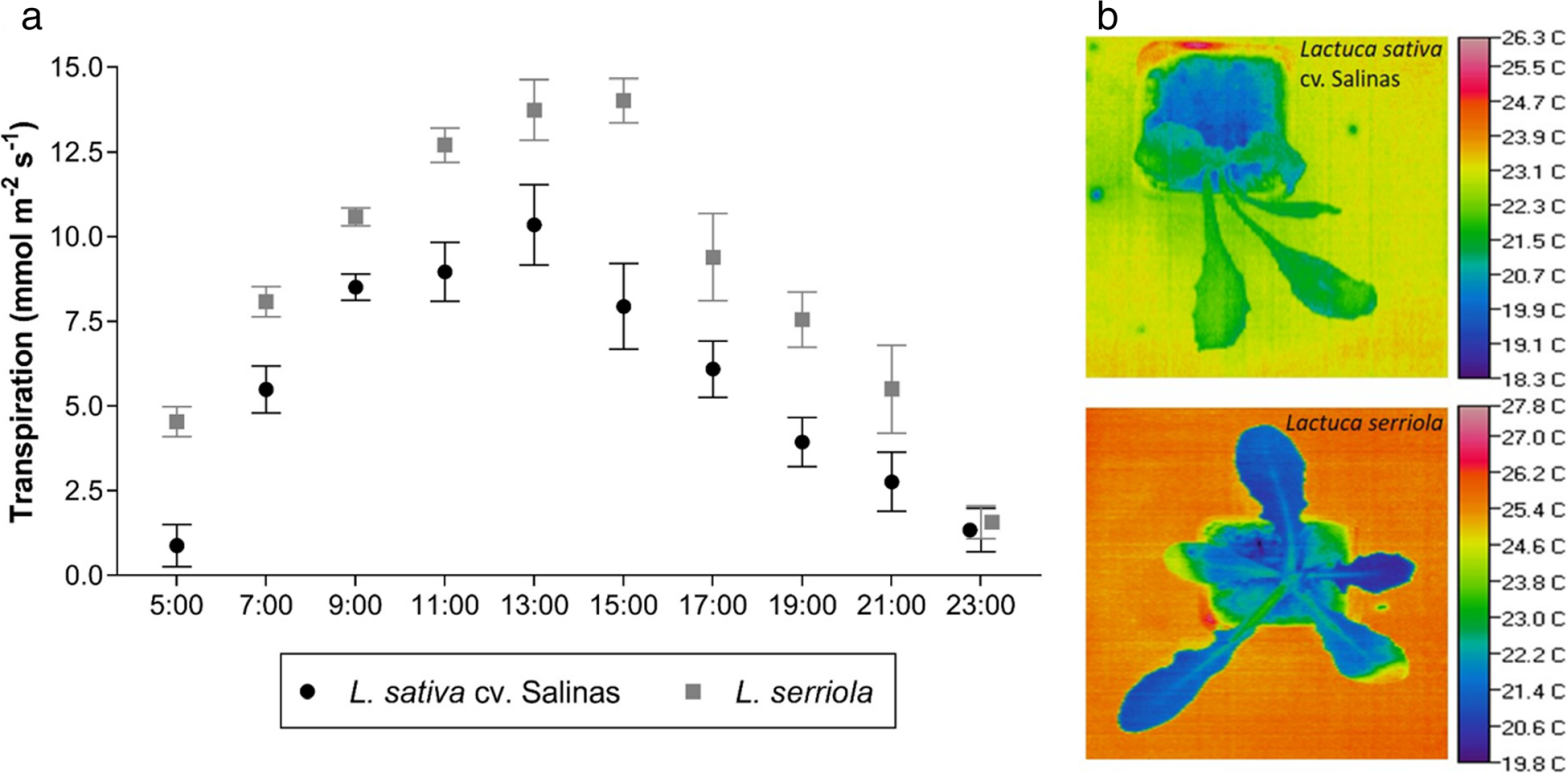
Diurnal transpiration of cultivated (*L. sativa* cv. Salinas) and wild (*L. serriola*) lettuce. Transpiration pattern (mmol m^− 2^ s^− 1^) (**a**), with example thermal images of cultivated and wild lettuce (**b**)

When grown under well-watered conditions, the wild (*L. serriola*) and cultivated (*L. sativa*) parents of the recombinant inbred lines (RILs) showed significant variation in their diurnal pattern of transpiration (repeated measures ANOVA; F_1,9_ = 24.76, *P* < 0.001, Fig. 1). For cultivated lettuce, transpiration rose from 05:00 until 13:00 h when it declined until measurements ceased at 23:00 (Fig. 1). Transpiration continued to rise until 15:00 h for wild lettuce, which demonstrated a significantly higher transpiration rate than its cultivated relative consistently throughout the course of the day under well-watered conditions (F_1,9_ = 24.76, *P* < 0.001, Fig. 1) until 23:00 h. This effect was observed in several experiments (data not shown).

**Fig. 2:**
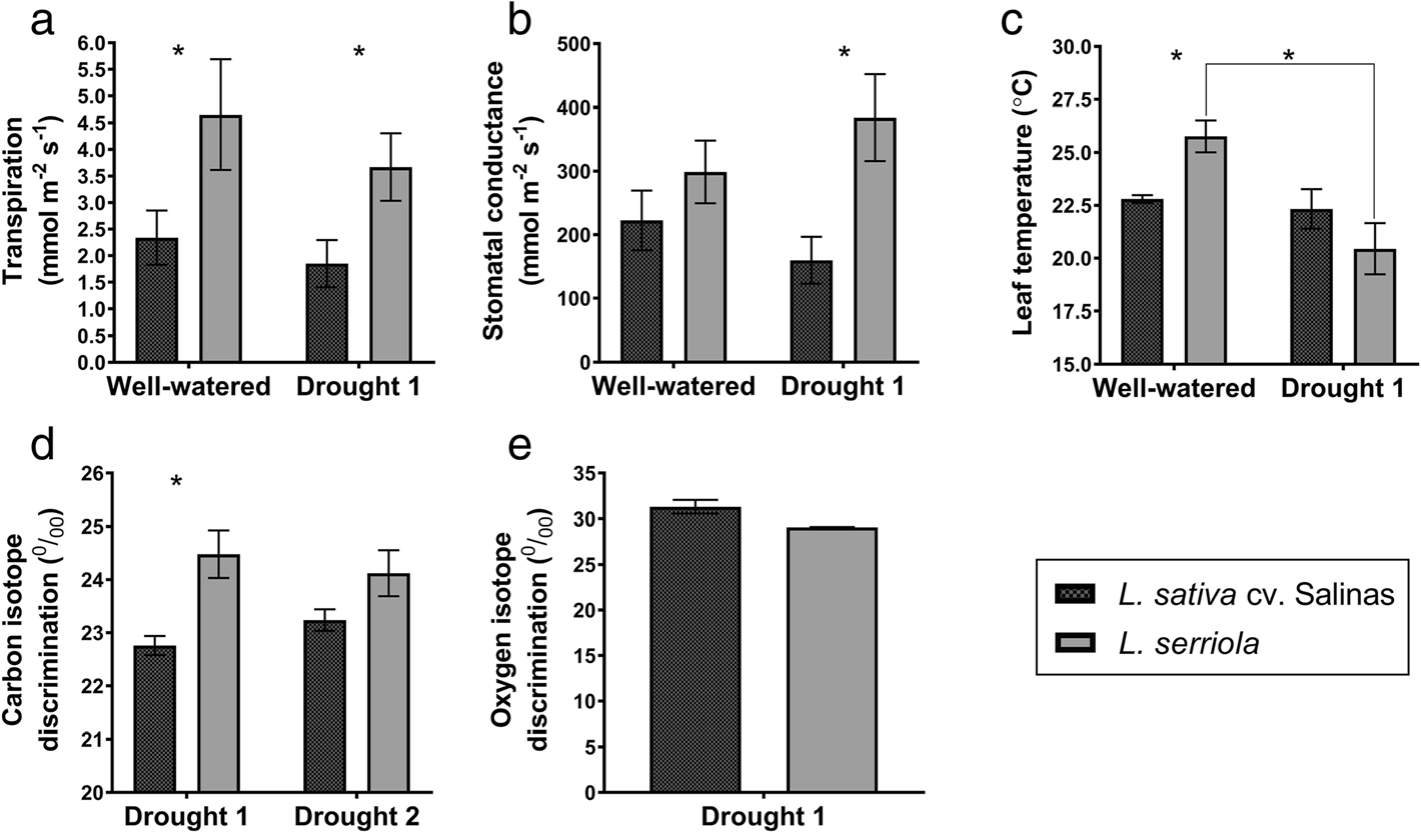
Drought response of cultivated (*L. sativa* cv. Salinas) and wild (*L. serriola*) lettuce. Transpiration (**a**), stomatal conductance (**b**), leaf temperature (**c**), carbon isotope (**d**) and oxygen isotope discrimination (**e**). * indicate significant differences (see text for details)

Transpiration rate was also higher in wild lettuce under drought (t_10_ = -2.35, *p* < 0.05, Fig. 2a) as was stomatal conductance (t_10_ = -2.90, *p* < 0.05, Fig. 2b). Although leaf temperature did not vary significantly between the two parents, there was a trend for lower leaf temperatures in wild lettuce when compared to the cultivated parent under drought (Fig. 2c), confirming the data from stomatal conductance. Leaf temperature was significantly higher in wild lettuce under well-watered conditions (t_10_ = -3.83, *p* < 0.01, Fig. 2c). Though differences between wild and cultivated lettuce were observed, the gas exchange response of both genotypes was negligible when the well-watered and drought 1 experiments were compared for each individual (Fig. 2a–c), however leaf temperature was significantly reduced by imposing water stress for wild lettuce (t_10_ = 3.73, *p* < 0.01, Fig. 2c). Carbon isotope discrimination (Δ^13^C) was consistently higher for wild lettuce compared to cultivated lettuce (Fig. 2d). Oxygen isotope discrimination was higher in cultivated lettuce (31.31 ± 0.75) than wild (29.08 ± 0.03), although differences were not significant.

### Phenotypic variation for water‐use traits in the RIL population

Phenotypes for water-use traits segregated under well-watered, mild and moderate drought conditions within the RIL population and bidirectional transgressive segregation was evident for transpiration, stomatal conductance, leaf temperature, fresh and dry weight (Figure S1). The RILs demonstrated transgressive segregation below either parent for carbon isotope discrimination under drought, indicating this population may have an improved water-use efficiency under these conditions.

**Fig. 3:**
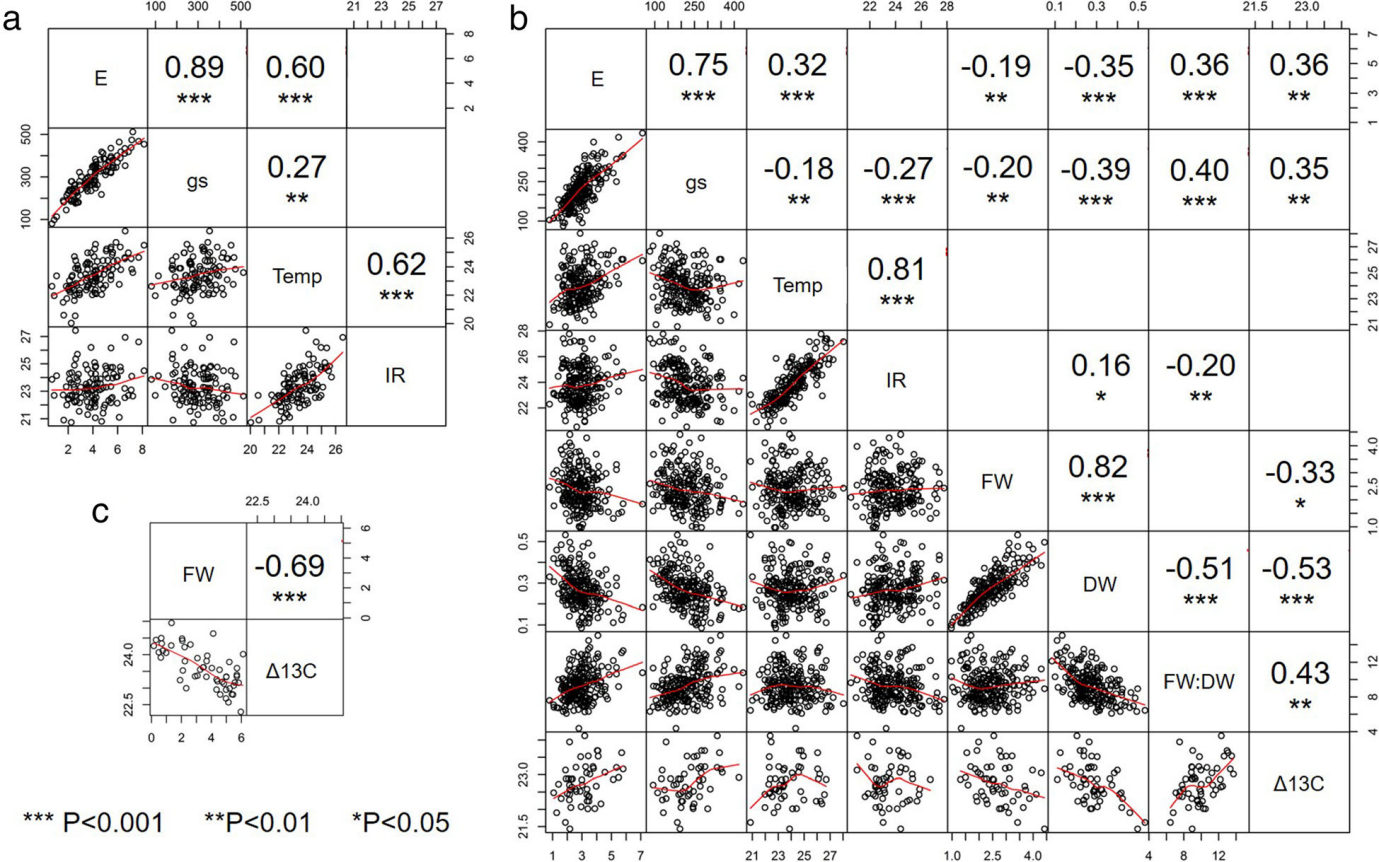
Correlations between water-use traits. Observed under well-watered conditions (**a**) and under drought 1 (**b**) and 2 (**c**) trials. Estimated using Spearman’s correlation, with scatterplot (bottom left) and significant r^2^ correlation values (top right) shown. * indicates significance at *P* > 0.001 (***), *P* < 0.01 (**) and *P* < 0.05 (*). Transpiration (**e**), stomatal conductance (g_s_), temperature measured by porometry (Temp), temperature measured by thermal imaging (IR), carbon isotope discrimination (Δ^13^C), whole fresh weigh (FW), dry weight (DW) and their ratio (FW:DW)

Infrared thermal measurements of leaf temperature correlated well with porometry measurements under well-watered (r^2^ = 0.62, *p* < 0.001, Fig. 3a) and drought conditions (r^2^ = 0.81, *p* < 0.001, Fig. 3b). Transpiration (E) was strongly positively correlated with stomatal conductance (g_s_) under well-watered (r^2^ = 89, *p* < 0.001, Fig. 3a) and drought conditions (r^2^ = 0.75, *p* < 0.001, Fig. 3b). Both E and g_s_ were significantly negatively correlated with fresh (r^2^=-0.19 and r^2^=-0.20, respectively, *P* < 0.01) and dried whole plant biomass (r^2^=-0.35 and r^2^=-0.39, respectively, *P* < 0.001), but positively correlated with fresh:dry weight ratio (r^2^ = 0.36 and r^2^ = 0.40, respectively, *P* < 0.001) in the Dr1 trial, although significant variation was observed in the data. Application of drought led to a reduction in g_s_ measured using a porometer (r^2^=-0.18, *p* < 0.01, Fig. 3b) and by thermal imagery (r^2^=-0.27, *p* < 0.001, Fig. 3b), though the opposite effect was seen under well-watered conditions when temperature was measured using the porometer (r^2^ = 27, *p* < 0.01, Fig. 3a). As expected, carbon isotope discrimination was found to be significantly negatively correlated with above ground fresh weight biomass under both drought treatments (r^2^=-0.33, *P* < 0.05, Fig. 3b and r^2^=-0.69, *P* < 0.001, Fig. 3c, for Dr1 and Dr2 treatments, respectively). Carbon isotope discrimination was positively correlated with E and g_s_ under drought stress (r^2^ = 0.36 and r^2^ = 0.35, respectively, *P* < 0.01, Fig. 3b).

### QTL for water‐use traits in lettuce

**Fig. 4:**
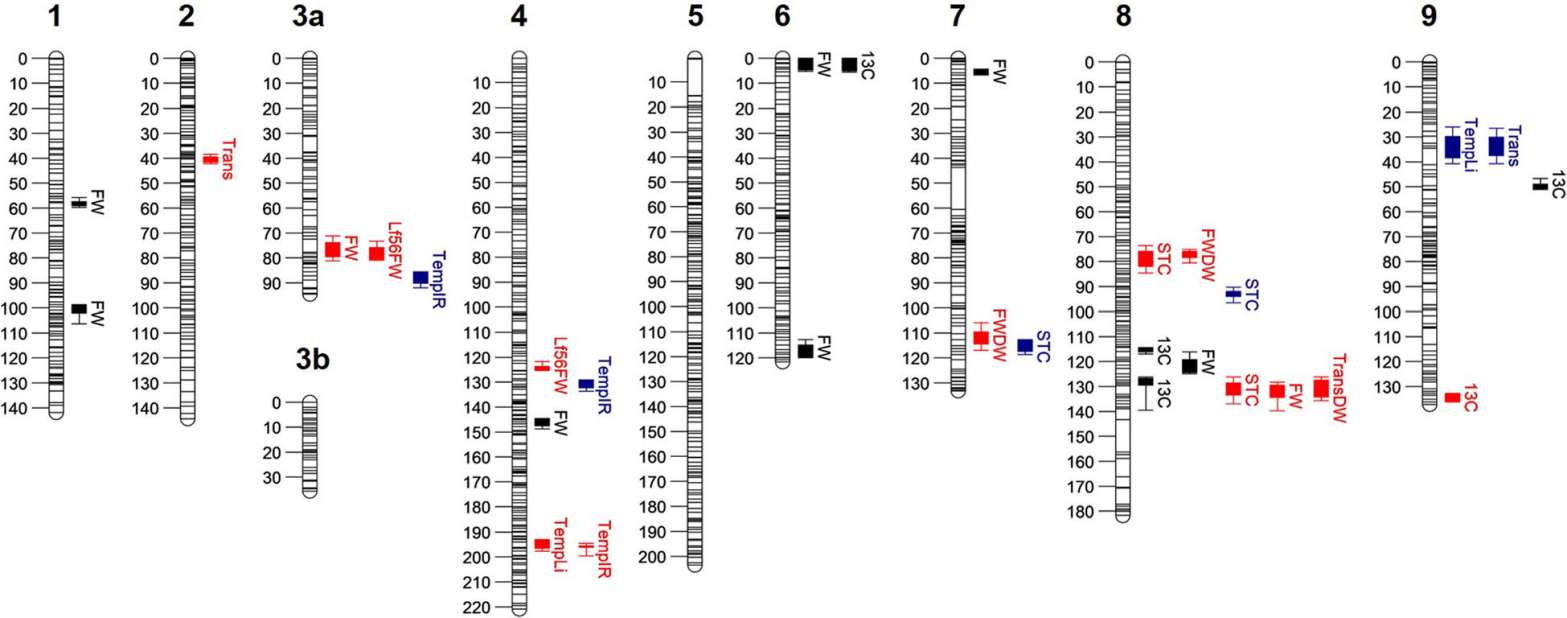
QTL identification for water-use-associated traits in the RIL population. Bars represent each LG with position in centiMorgan on the left, LG number at the top of each bar and horizontal lines indicating marker positions. QTL are shown as filled boxes to the right of each LG representing the 1-LOD interval, with error bars showing the 2-LOD interval for QTL detected in the well-watered (blue), Dr1 (red) and Dr2 (black) trials. 13 C, Δ^13^C, FW, fresh weight; FW_ Lf56, fresh weight of fifth and sixth true leaves, FW:DW, fresh:dry weight ratio; gs, stomatal conductance, E, transpiration, E:DW, transpiration:(dry weight) ratio; Temp, leaf temperature measured via porometry, IR, leaf temperature measured via IR thermography

A genetic linkage map with 1,099 markers spanning a total of 1,414.7 cM across 10 linkage groups was generated using regression mapping in Joinmap. Collinearity of marker ordering was validated using the physical map. Due to a region of high segregation distortion on chromosome 3, which has been previously noted by Truco et al. [7], this linkage group did not coalesce and was split into two segments labelled as 3a and 3b, which were 62 and 33 cM in length, respectively. Maximum marker interval was 16.9 cM with an average spacing of 1.3 cM (approximately 2.2 Mb) across all LGs. Utilising this molecular marker map, 30 significant QTL were identified for nine of the ten traits investigated, with no QTL identified for whole plant dry weight. These QTL accounted for 4.8–23.6 % of the phenotypic variation (PV), with 22 small effect QTL (< 10 % PV) and eight moderate effect QTL (10–25 % PV) and spanned eight of the ten linkage groups, with no QTL identified on LG5 or LG3b (Table 1; Fig. 4).

**Table 1:** QTL detected in the RIL population through composite interval mapping (*p*<0.05)

Trial	Trait	LG ^**a**^	QTL ID ^**b**^	Position (cM) ^**c**^	Position (Mbp) ^**d**^	LOD^**e**^	PVE (%) ^**f**^	Additive ^**g**^	Allele ^**h**^	H^**2 i**^
WW	Stomatal conductance	7	qSTC7.1	115.1	164.70	3.59	6.78	0.53	*L. serriola*	0.22
		8	qSTC8.2	92.9	124.67	5.28	10.39	0.53	*L. sativa*	
	Transpiration	9	qTra9.1	32.5	48.76	3.35	7.51	0.44	*L. sativa*	0.17
	Temperature (Por)	9	qTPr9.1	33.8	50.50	3.60	9.07	0.39	*L. sativa*	0.06
	Temperature (IR)	3a	qTIR3a.1	87.2	125.48	4.24	8.84	0.49	*L. serriola*	0.08
		4	qTIR4.1	130.6	184.82	3.75	8.12	0.47	*L. serriola*	
Dr1	Stomatal conductance	8	qSTC8.1	76.6	95.92	3.49	5.51	0.32	*L. sativa*	0.00
		8	qSTC8.3	129.6	192.88	3.23	5.05	0.32	*L. serriola*	
	Transpiration	2	qTra2.1	39.8	85.13	5.14	8.39	0.31	*L. sativa*	0.00
	Transpiration:dry weight	8	qTDW8.1	129.6	192.88	4.05	6.61	0.29	*L. serriola*	0.11
	Temperature (Por)	4	qTPr4.1	195.8	327.97	3.40	5.86	0.25	*L. serriola*	0.00
	Temperature (IR)	4	qTIR4.2	195.7	327.97	5.56	9.54	0.27	*L. serriola*	0.02
	Δ13C	9	q13C9.2	137.2	201.14	3.67	5.96	0.3	*L. sativa*	0.54
	Fresh weight (leaves 5 + 6)	3a	qFWl3a.1	78.2	102.91	5.43	8.32	0.35	*L. sativa*	0.00
		4	qFWl4.1	125	176.29	3.24	4.85	0.35	*L. sativa*	
	Fresh weight (whole)	3a	qFWw3a.1	78.2	102.91	4.67	7.62	0.3	*L. sativa*	0.32
		8	qFWw8.2	133.1	196.57	3.48	5.59	0.3	*L. sativa*	
	Fresh:dry weight (whole)	7	qFDW7.1	110.3	161.40	4.28	6.74	0.33	*L. serriola*	0.36
		8	qFDW8.1	76.6	95.92	5.41	8.53	0.33	*L. sativa*	
Dr2	Δ13C	6	q13C6.1	3.6	7.51	7.94	23.57	0.76	*L. serriola*	0.54
		8	q13C8.1	115.4	170.46	3.56	9.34	0.77	*L. serriola*	
		8	q13C8.2	128.3	188.29	5.76	15.38	0.76	*L. sativa*	
		9	q13C9.1	49	65.22	5.50	15.15	0.77	*L. serriola*	
	Fresh weight (whole)	1	qFWw1.1	57.9	63.24	5.77	10.04	0.85	*L. sativa*	0.55
		1	qFWw1.2	100.9	130.13	6.65	12.07	0.85	*L. serriola*	
		4	qFWw4.1	145.6	224.71	8.41	16.60	0.85	*L. serriola*	
		6	qFWw6.1	3.6	7.51	9.30	19.17	0.85	*L. sativa*	
		6	qFWw6.2	116.6	182.06	4.70	7.78	0.85	*L. serriola*	
		7	qFWw7.1	5.8	14.05	5.03	8.54	0.85	*L. sativa*	
		8	qFWw8.1	120.1	177.23	3.71	5.83	0.85	*L. serriola*	

^a^Linkage group number

^b^QTL ID according to lettuce convention (“q”, followed by three letter trait code, linkage group number and order of QTL within linkage group)

^c^QTL peak position in genetic linkage map, in centimorgans

^d^Physical QTL peak position in *Lactuca sativa* cultivar Salinas reference genome (V8), in Mb

^e^Logarithm of odds score

^f^Percentage of phenotypic variance explained by the QTL

^g^Additive genetic variance

^h^Allele responsible for increase in trait value

^i^Broad-sense heritability

Two QTL for E were identified on LGs 2 and 9, under the Dr1 and well-watered treatments and accounting for 8.4 and 7.5 % of the PV respectively, with *L. sativa* allele inheritance increasing the trait value. A QTL for leaf temperature measured via steady state porometry mapped to the same position as E on LG 9 accounting to 9.1 % of the PV. Four QTL for g_s_ were identified, two under the well-watered treatment on LGs 7 and 8, cumulatively accounting for 17.2 % of the PV and two under Dr1 treatment, located 53 cM apart and accounting for 10.6 % of the PV. Three moderate and one small-effect QTL for Δ^13^C measured in the Dr2 trial were identified on LGs 6, 8 and 9, together accounting for 63.4 % of the PV. QTL for Δ^13^C co-located to those for whole plant fresh weight (FW) on LGs 6 and 8 in the Dr1 trial and a second QTL for Δ^13^C on LG 8 located to the same position as FW, g_s_ and the ratio between E and whole plant dry weight measured in the Dr1 trial. Two QTL for leaf temperature, measured by porometry and IR thermal imaging in the Dr1 trial co-located on LG 4, accounting for 5.6 and 9.5 % of the PV. Mapping has identified interesting candidate regions for further functional investigations.

### Candidate genes for WUE in lettuce

Nine locations with large-effect or multiple overlapping QTL were selected for candidate gene analyses (Table S2). The 2-LOD QTL intervals were mined for genomic features, identifying > 1,400 putative genes from 73.8 Mbp of genome sequence and 87 % of these genes retrieved a BLASTp hit against 15 plant protein databases (Table S3).

**Fig. 5:**
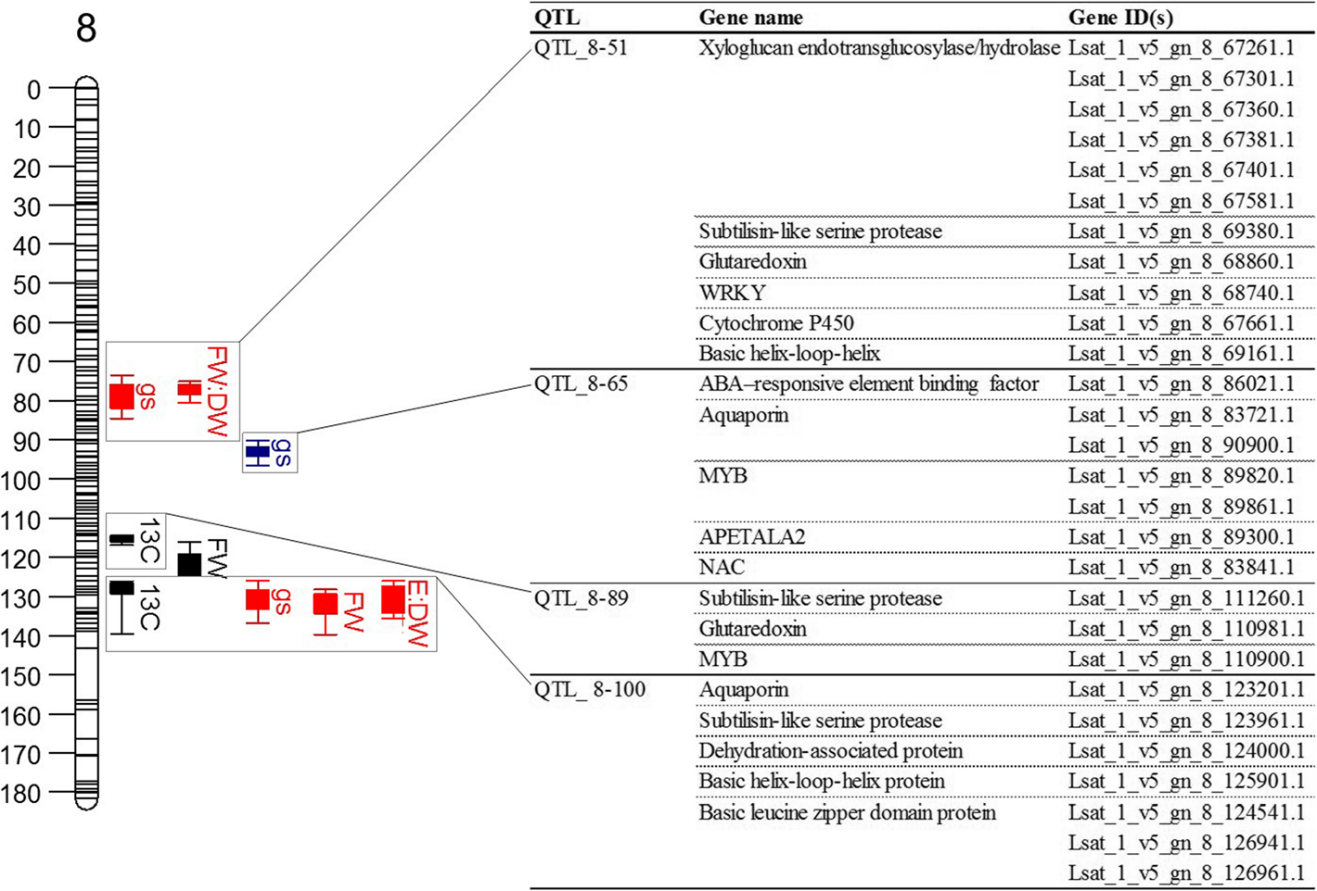
Candidate gene mining of LG8 QTL. Illustration of LG 8, with the QTL investigated for candidate genes highlighted and gene information provided

Four regions of interest were located on LG8 (Fig. 5). QTL_8–51, comprising two QTL for g_s_ and FW:DW, and these harboured a cluster of six xyloglucan endotransglucosylase/hydrolases (XTH) which have roles in modifying the extensibility of the cell wall and have been linked to drought tolerance through influencing stomatal pore size [42]. Other candidates in this region included a subtilisin-like serine protease, which modulate cell differentiation during stomatal development [43], a glutaredoxin family protein, associated with drought stress tolerance through ROS detoxification [44] WRKY and BHLH transcription factors. Significantly enriched GO terms within QTL_8–51 included those for cell wall (GO:0005618), cellular polysaccharide metabolic process (GO:0044264) and xyloglucan:xyloglucosyl transferase activity (GO:0016762; Table S4). A subtilisin-like protease and glutaredoxin family protein were identified within QTL_8–89, a QTL for Δ^13^C accounting for 9 % of the PV. A QTL for g_s_ accounting for 10 % of the PV on LG 8, QTL_8–65, mapped to the same position as two aquaporin-like proteins involved in water transport and an ABA-responsive element binding protein, involved in ABA-induced stomatal closure following water deficit [45]. Another aquaporin protein was identified within QTL_8-100; a hotspot on LG8 in which QTL for Δ^13^C, g_s_, FW and the ratio between E and DW co-located. Other notable candidates in this QTL hotspot included a subtilisin-like serine protease, a dehydration-associated protein, three BZIP and one BHLH transcription factors (Fig. 5). Significantly enriched GO terms within QTL_8-100 included defence response (GO:0006952), response to stress (GO:0006950), stimulus (GO:0050896), oxidative stress (GO:0006979) and antioxidant activity (GO:0016209; Table S4).

Under QTL_6–6, a region in which large-effect QTL for Δ^13^C and FW traits measured from the Dr2 trial co-located, a late embryogenesis abundant (LEA) protein along with several transcription factors reported to influence response to drought were identified, including three MYB-like domain containing proteins, a NAC, APETALA2 (AP2)-like ethylene-responsive factor and WRKY transcription factor (Hadiarto & Tran, [46]; Nuruzzaman et al., [47]; Table S3). The same region contained ten glutathione S-transferases and a glutathione peroxidase, involved in reactive oxygen species (ROS) detoxification in response to drought [48]. A region in which a QTL for E and leaf temperature co-localised, designated QTL_9–27, contained a LEA protein, an aquaporin-like protein, a glutathione S-transferase and several transcription factors (AP2, MYB, NAC, WRKYand BZIP; Table S3).

## Discussion

### Understanding the ideotype for improved water‐use efficiency in lettuce

Improving crop WUE in response to climate change is imperative; particularly for crops such as lettuce, with over 75 % of total production in the US dominated by the state of California, which most recently experienced a 7-year period of drought [49, 50]. We have identified QTL for water-use traits, including g_s_, E and Δ^13^C and mapped these alongside yield, in a critical key step towards elucidating the genetic basis of WUE. We have observed key differences in these water-use traits in wild and domesticated lettuce.

Generally, wild lettuce had increased stomatal conductance, transpiration rate and carbon isotope discrimination compared to cultivated lettuce (Fig. 2), all of which indicate a reduced WUE. Stomatal density was previously measured to be up to 30 % higher in wild lettuce compared to cultivated, which is likely to at least partially explain the reduction in WUE [16]. Wild lettuce also had a reduced whole plant fresh weight compared to cultivated lettuce in the Dr1 trial and the effect was more dramatic at over two thirds in the Dr2 trial (Figure S1), consistent with previous investigations [16]. The lower fresh weight of wild lettuce reflects the differences in plant morphology, with leaf architecture drastically altered through domestication from the long, thin, serrated leaves of wild lettuce to broad, circular leaves observed in the lettuce cultivar, which have been measured to have an increased leaf area of up to 40 % [16]. Wild lettuce has been shown to display differences in root architecture, with a longer taproot enabling water utilisation from deep soil layers, whilst cultivated lettuce has a shallow root system [8]. Indeed, QTL for taproot length have been found to coincide with deep soil water exploitation [12]. This is unsurprising, given that this accession of wild lettuce was collected from a dry region where a greater water reserve is deeper below the surface, whilst cultivated lettuce is exposed to ample water and fertiliser applied at the soil surface. An enhanced ability to utilise water from deep soil layers may explain the higher rate of transpiration throughout longer periods of the day as observed for wild lettuce, even under drought. However, differences between wild and cultivated lettuce suggest that wild lettuce had a lower WUE, despite these root characteristics. Wright et al. [51] highlighted the importance of considering both shoot and root Δ^13^C when estimating WUE, with differences in root architecture found to alter this relationship. Research has indicated that juvenile pre-flowering plants may shift from low water-use efficiency (high Δ^13^C) to high water-use efficiency (low Δ^13^C) after establishment [52, 53]. This strategy could promote a greater carbon investment early in the life cycle, enabling the development of deeper roots and the accumulation of biomass whilst water is more abundant and plants are smaller, then switching to higher WUE once plants are established and water is scarce [52]. Such a strategy may be utilised by wild lettuce, which is adapted to germinate during wetter spring conditions and flowers during summer drought, whereas cultivated lettuce is adapted to a consistently well-watered environment.

### Future breeding of lettuce with improved WUE

Carbon isotope discrimination (Δ^13^C) has previously been shown to be an accurate indicator of WUE [54], with reduced Δ^13^C indicating an increased WUE, and has been linked with grain yield in cereal crops including wheat [55], barley [56] and rice [57]. In the Australian wheat breeding program, low Δ^13^C lines were crossed with a high yielding cultivar to develop ‘Drysdale’ and ‘Rees’ [22]. These novel varieties out-yielded commercial lines in drought conditions, with an increase in yield of 2–15 % in low Δ^13^C lines compared to high lines and the highest improvements occurring in the most droughted conditions. Identification of transgressive segregants for Δ^13^C in the lettuce RIL population, with trait values below that of either parent, therefore provides critically important genetic material for future breeding programs. QTL for WUE have mapped alongside those for Δ^13^C in the C3 plant sunflower [58] and C4 grass Setaria [59] and QTL for Δ^13^C in wheat have been found repeatedly across multiple environments [60]. In this study, we identified three instances of QTL for Δ^13^C mapping alongside fresh weight yield under drought stress and this trait had some of the highest values of broad-sense heritability that we recorded, adding further weight to the use of this trait as a proxy for WUE. In particular, in a cluster on LG 8, QTL for stomatal conductance, fresh weight and the ratio of transpiration:dry weight mapped to the same position as Δ^13^C under drought conditions, providing a genomic region associated with water-use traits in lettuce of interest for further exploration.

Notably in this study, QTL variation with environment change is evident, with genotype effects being modulated by the environment (well-watered, Dr1 and Dr2 trials) resulting in phenotypic plasticity. The result of this is QTL for a given trait often do not appear consistently under different treatments, highlighting the complex genotype by environment (GxE) interactions of water-use traits. Hartman et al.[6] subjected the core population utilised in the present investigation to drought, low nutrient, high salinity and competition stressors and noted little co-location of yield QTL between different trials. Environmentally-induced stomatal closure due to changes in water availability have a GxE effect, reducing heritability of the trait [61]. This was reflected in the broad-sense heritability estimates for traits including E and g_s_, which demonstrated a reduced H^2^ in the Dr1 trial compared to the well-watered trial and likely explains why QTL for this traits did not map to the same location in different trials. Interestingly, Δ^13^C heritability was consistent in drought trials, but no co-locating QTL were identified, highlighting the complex nature of this trait. Therefore, identifying robust molecular markers that are informative across different environments remains challenging.

The potential for using the genetic diversity offered by wild crop relatives in order to improve commercial crops has been well documented [62] and it may be possible to utilise a similar approach to make genetic gains in lettuce. In baby leaf lettuce breeding, cultivar development should aim to produce a line which performs normally under well-watered conditions, whilst being able to maintain yield and quality if water deficits occur. QTL identified for fresh weight under drought with wild alleles responsible for an increase in trait value contributed to 42.3 % of the PV, providing novel alleles for future breeding efforts to increase yield under abiotic stress.

Plants may deploy contrasting strategies in response to drought stress: escape, avoidance or tolerance and many breeding approaches to improve crop drought avoidance and tolerance, are possible. These include reducing transpiration through decreased leaf growth and stomatal closure through hormone action, modifying transcriptional regulation, targeting molecules, such as osmoprotectants, which stabilise cell biochemical reactions (for example, proteins, membranes and biological structures affected by dehydration) or that prevent toxic molecules accumulating as a by-product of ROS accumulation [63]. Here, several genes with established roles in influencing WUE and drought response were found to be collocated with QTL. Two LEA proteins were identified within overlapping QTL intervals of Δ^13^C and FW, and transpiration and leaf temperature. LEA proteins are a large family of non-enzymatic proteins, first observed to accumulate in the later stages of seed development prior to desiccation and were later found in vegetative tissues under dehydration stress [63]. Aquaporins, conserved membrane water channels, were also identified within two independent QTL for g_s_ and one for E. Essential for water homeostasis, aquaporins are generally downregulated in response to drought stress [64].

Other important candidates, identified within overlapping QTL intervals for Δ^13^C and FW and g_s_ and FW:DW were the cell wall remodeling enzymes, XTHs [65]. Overexpression of XTHs has been found to increase stress tolerance in *Arabidopsis* and tomato, hypothesised to result from changes in cell wall extensibility influencing stomatal aperture [42]. Subtilisin-like serine proteases, identified in the present study within two QTL for g_s_, were first linked to stomatal development after identification of an *Arabidopsis* mutant with > 3-fold increase stomatal density and irregular patterning [43]. Antioxidant detoxification and osmotic adjustment are key mechanisms of stress tolerance following prolonged drought. Glutathione S-transferases, identified within QTL for Δ^13^C, are enzyme antioxidants which demonstrate increased expression in response to abiotic stress and overexpression in transgenic *Arabidopsis* was shown to improve osmotic stress tolerance [66]. Similarly, glutaredoxins are glutathione-dependent reductases involved in maintaining redox balance following drought stress, with silencing found to reduce relative water content in tomato [44]. Targeting WUE through enhancing antioxidant detoxification following stress is unlikely to have the same negative impacts on yield as methods which modify leaf physiology and is an important target for improving WUE in lettuce. Finally, the role of transcription factors in drought response through the regulation of the abscisic-acid (ABA)-dependent and ABA–independent signal transduction has been extensively reviewed (for example, ref. [45]). During the ABA-dependent signaling response, accumulation of the phytohormone ABA in response to water deficit leads to stomatal closure through interaction with ABA-responsive element binding proteins [45], which we identified within a QTL interval for stomatal conductance on LG 8. ABA is also known to modulate MYB, WRKY, BZIP and NAC transcription factors [45], a combination of which were identified within all but one of the candidate QTL regions investigated.

Whilst the candidates described here provide an important starting point into the exploration of the genetic basis of water use in lettuce, an important next step will be to analyse expression relationships and further refine and fine map these loci using BSA-Seq and other fine mapping approaches [67]. It is also significant to note that QTL are not always consistent across different genetic backgrounds, highlighting the importance in validating functional relationships between QTL and candidate genes. Plant biotechnology provides an attractive approach to elucidating the genetic regulation of WUE by functional analyses. One of the difficulties in such approaches, however, is the established link between water use and plant productivity. Wheat genetically modified to express an ABA-responsive barley gene, *HVA*1, had significantly higher WUE than the wild type under water deficit conditions with improved biomass productivity [68]. Maintaining yield in leafy crops such as lettuce, where the harvest index is vegetative, is perhaps more complicated, however these successes provide optimism that progress can be made using biotechnology, given the vast genomic resources at our disposal [69]. Success of such approaches in revealing more about the genetic regulation of WUE are likely to be quickly advanced with emerging genome editing tools. Using genome editing technologies, rapid, targeted and highly precise gene modifications can now be made [70]. CRISPR-Cas systems have been successfully implemented to explore drought tolerance in a variety of crops, including maize [71], tomato [72] and rice [73] and in future can be exploited in lettuce, targeting candidates identified here with the aim of validating the involvement of these genes in determining WUE of this important leafy crop.

### Application of leaf thermography in lettuce breeding

Within the last ten years crop canopy temperature, as detected using thermal imaging, has been identified as a powerful tool for precision irrigation scheduling [33] and in the present investigation, IR images of leaf temperature correlated well with direct thermocouple measurements, suggesting that this rapid, non-invasive technology has potential for use in lettuce irrigation scheduling. Thermal imaging can also be employed as a screening target for breeding for abiotic stress tolerance [33]. Work is on-going to confirm the ability to predict yield capacity using canopy temperature in different environmental scenarios. If thermal imaging can be used to screen for yield in commercial breeding programs of all crop types, traditional phenotyping would be revolutionised, with drastic reductions in the time required to assess populations and therefore breed drought tolerant plants. Quantitative genetic investigation of this trait has been limited, with a large focus on rice and wheat [74–76]. These studies have yielded robust QTL for canopy temperature, which also controls grain yield, and fine mapping is being used to determine the genes which underlie the genetic control of these traits [76]. We have identified QTL for leaf thermography under well-watered and drought conditions, which mapped alongside QTL for whole plant fresh biomass on LGs 3a and 4, identifying a link between canopy temperature and yield in lettuce. Concurrent mapping of QTL measured via steady state porometry and IR imaging on LG4 along with a strong positive correlation in measurements under well-watered and drought conditions suggests thermography could be an accurate, instantaneous and indirect measure of leaf temperature. The investigation of the genetic basis of canopy temperature in lettuce, a non-grain food crop, as presented for the first time here, is a crucial step towards determining whether yield in this leafy crop can be predicted in the same way as for grain crops.

## Conclusions

Understanding and improving the WUE of irrigated crops such as lettuce is imperative if we are to select and breed crops resilient to future water-limited environments. In this study, we utilised a quantitative genetics approach to unravel the genetic architecture of lettuce WUE. Key traits underpinning WUE, including carbon isotope discrimination and stomatal conductance, were measured alongside yield traits, under control, well-watered and two water-limiting treatments. For the first time, we identified QTL for WUE traits in lettuce, identifying regions where QTL for yield mapped alongside carbon isotope discrimination and stomatal conductance. At the same time, we demonstrated the feasibility of using remotely sensed leaf temperature (IR) data to quantify plant water-use, as a rapid and non-destructive technique that may be used in large screening programs, with IR positively correlated with biomass yield. Our analyses revealed promising candidate genes for further exploration.

## Methods

### Plant material

An existing mapping population of 209 F_9 − 10_ RILs, derived from *L. serriola* (US96UC23) and *L. sativa* cv. Salinas were utilised in this study, developed in the Michelmore laboratory as described in ref. [7]. *L. serriola* (US96UC23) seed was identified and collected from public land in Davis, California by the Michelmore group and the cultivar Salinas was developed and distributed by Edward J. Ryder at the US Agricultural Research Service. This population is a reference mapping population being studied for numerous traits by groups worldwide. An experimental summary of each trial can be viewed in Table S5. In the first of three glasshouse trials (July, 2009), referred to as the well-watered trial (WW), a subset of 122 RILs were assessed. Plants were watered by flood benching when required according to commercial standards and maintained under 16 h day length (supplementary lighting supplied from 06:00–22:00) with average day and night temperatures of 18 and 14 °C, respectively, before harvest at 42 d. In the second trial (August, 2009), referred to as the drought 1 trial (Dr1), the complete population (209 lines) was assessed. Plants were watered by flood benching as required for the first 26 days of growth, after which soil moisture was reduced to 10–20 % volumetric water content, as measured daily using a Delta-T ML2x ThetaProbe connected to an HH2 moisture meter (Delta-T Devices, Cambridge, UK). Plants were maintained under 16 h day length (supplementary lighting supplied from 06:00–22:00) with average day and night temperatures of 18 and 14 °C, respectively, before harvest at 40 days. In the third trial (June, 2012), referred to as drought 2 trial (Dr2), a subset of 60 of the most informative RILs were assessed, based on the number of recombination break points [16]. Plants were watered by flood benching as required for the first 26 days of growth, after which water was completely withheld until harvest at 32 days. Plants were maintained under 16 h day length (supplementary lighting supplied from 07:00–19:00), with day and night temperatures averaging 18 and 15 °C, respectively. The trial layout for each treatment (WW, Dr1, Dr2) was fully randomised and included three replicates of each RIL and six replicates of each parent line. One seed was planted per 7.5 cm^2^ pot (approximately 0.25 L volume) in a 1:1 mixture of vermiculite (Sinclair, UK) and Humax professional range nursery stock compost (Humax, UK), with two rows of guard plants (green romaine) surrounding each trial.

### Trait data collection

Abaxial stomatal conductance was measured on the fifth true leaf, where leaf 1 was the oldest and leaf 6 was the youngest leaf. Measurements of transpiration (E), stomatal conductance (g_s_) and leaf temperature were taken using a porometer LI-1600 (Li-cor, Nebraska, USA). Leaf 5 was imaged at a 90° angle at approximately 1 m height using an infra-red camera TH9100WR (NEC, Metrum, Tokyo, Japan), which operated in the region of 8–14 μm with 0.1 °C thermal resolution and a spatial resolution of 320 (V) x 240 (H) pixels. Emissivity was set at 1.0 as it has been reported to induce errors of less than 1 °C [77]. Air temperature was logged continuously using Testo 174 data loggers (Testo, Alton, UK).

Total above ground fresh biomass was assessed using a top loading Sartorius Analytic balance (AC120S, Sartorius, New York, USA) after excision at the stem base. Biomass was oven dried at 80 ºC in paper bags to achieve constant mass, before dry weight measurement and determination of plant fresh to dry weight ratio. The fifth and sixth leaves were consistently identified and used for carbon isotopic discrimination analyses from both of the mild and moderate drought trials, on a subset of the sixty most informative RILs based on genetic diversity [16], with three replicates per genotype. Dried leaves were ground to a fine powder using a mixer mill (MM300, Retsch, Haan, Germany), before 0.3–0.4 or 0.2 mg of ground material was inserted into a 6 × 4 mm tin or silver capsule for carbon or oxygen isotope analysis, respectively (SerCon, UK). For carbon isotope analysis, samples were analysed using a Flash 1112 elemental analyser (EA) coupled to a Delta V Advantage isotope ratio mass spectrometer (IRMS; Thermo-Fisher, Bremen, Germany) and were introduced into the EA by a solid autosampler. The reactor tubes of the EA were self-packed with two quartz glass tubes filled with chromium oxide/copper oxide and reduced copper for combustion and reduction, respectively. Combustion and reduction reactor temperatures were 1,020 and 640 °C, respectively, while the post-reactor GC column was kept at 35 °C for separation of evolved CO_2_ (Meier-Augenstein, 2011, personal communication). Oxygen isotope analysis was conducted using a DeltaPlus-XP IRMS coupled to High Temperature Conversion/Elemental Analyser (Thermo-Fisher, Bremen, Germany) and were introduced into the EA using a Zero-Blank solid autosampler. The reactor tube was self-packed with an outer AlsintTM ceramic tube and an inner glassy carbon tube (Sigradur®, HTW, Thierhaupten, Germany) filled with glassy carbon granulate, silver and quartz wool (SerCon, UK). Reaction temperature was set to 1,425 °C and the post-reactor GC column was maintained at 85 °C.

The carbon isotope composition of the sample was determined using the following formula:

$$ {\delta}^{13}\mathrm{C}\left(\raisebox{1ex}{$0$}\!\left/ \!\raisebox{-1ex}{$00$}\right.\right)=\left[\left({\mathrm{R}}_{\mathrm{sample}}/{\mathrm{R}}_{\mathrm{reference}}\right)/{\mathrm{R}}_{\mathrm{reference}}\right]\times 1000 $$

where R_sample_ and R_reference_ are the ^13^ C/^12^ C ratio of the sample and reference, respectively [39].

Further, Δ^13^C was calculated using the following formula:

$$\bigtriangleup=\left({\mathrm\delta}_{\mathrm a}-{\mathrm\delta}_{\mathrm p}\right)/\left[1+\left({\mathrm\delta}_{\mathrm p}/1000\right)\right]$$

Where δ_a_ and δ_p_ are the carbon isotope composition of the air, which has been approximated at about − 8 ‰ [54], and the plant sample, respectively.

Oxygen isotope composition was calculated using the following formula:

$$\mathrm\delta^{18}\mathrm O={\mathrm R}_{\mathit p}/{\mathrm R}_{\mathit s\mathit t}-1$$

R_p_ is the isotope ratio of the plant and R_st_ is the isotope ratio of the standard, which in the case of oxygen isotope analyses is the Vienna-Standard Mean Oceanic Water (2.0052 × 10^− 3^).

### Data analyses

Statistical analyses were conducted using R Statistics (R core team, 2017). Trait distribution was assessed by examining skewness and kurtosis and normality was assessed using the Shapiro-Wilk test, with data normalised using log or reciprocal transformation as necessary. Diurnal patterns of transpiration for both parents were analysed using a repeated measure analysis of variance (ANOVA) with genotype and time as factors. Broad-sense heritability (H^2^) was estimated form one-way ANOVA as described in Iraqi et al. [78]. Parental means were compared using two-sample t-tests. All thermal imagery was analysed using Image Processor Pro II software (Version 4.0, NEC, Tokyo, Japan). Graphs were drawn in using GraphPad Prism version 7 (GraphPad Software, La Jolla, CA, USA). Spearman’s rank correlation coefficient was calculated and visualised using the ‘PerformanceAnalytics’ package in R.

### Linkage map construction and QTL mapping

For genotyping, DNA extraction, GBS library preparation and sequencing and GBS data processing were performed as described [79]. GBS data was aligned to the *L. sativa* reference genome (V8) and SNPs were called using the TASSEL 3.0 GBS pipeline, with the parameters described [79]. In TASSEL 5.0, SNPs were filtered to remove any that were homozygous between the parental genotypes and all heterozygous genotypes were set to unknown. Data were imported to R statistics for further filtering to remove SNPs with > 10 % missing data and those with significant segregation distortion using the r/qtl package [80]. Redundant SNPs were eliminated using the BIN function in QTL IciMapping [81]. A dense genetic linkage map was constructed from the remaining SNPs using the regression algorithm and Kosambi mapping function in Joinmap 4.0 (Kyazma, Netherlands).

QTL analysis was implemented in QTL Cartographer 2.5 (North Carolina State University, NC, USA) using composite interval mapping on averaged, normally distributed RIL phenotype data. Ten markers were selected to control for background genetic variation by forwards stepwise regression with backwards elimination and incorporated into the statistical model as cofactors. A window size of 10 cM flanking the test site was applied and the interval between test sites was 1 cM. The logarithm of odds (LOD) threshold for QTL significance at α = 0.05 was determined for each trait by permutation with 1000 iterations.

### Candidate gene mining

Candidate genes underlying QTL clusters of interest were identified from the overlapping regions of the 2-LOD support intervals (region of the LOD curve in which the LOD score is within 2 of the maximum). Markers closest to the 2-LOD interval were identified and all annotated coding sequences (CDS) within this genomic region were retrieved from the *Lactuca sativa* V8 genome (genome ID: 35,223) via the CoGe Comparative Genomics platform [82]. Protein CDS were used in a BLASTp search against the peptide sequences of 15 plant genomes (Supplementary Table S1) accessed from Ensembl Plants (http://plants.ensembl.org/index.html), with an E value cut-off of < 1 × 10^− 5^, using BLAST + 2.8.1 (https://ftp.ncbi.nlm.nih.gov/blast/executables/blast+/). Gene ontology (GO) enrichment analyses of the candidate QTL regions was completed using the singular enrichment analysis tool on agriGO v2.0 [83], at *P* < 0.05 significance threshold with Bonferroni correction.

## Supplementary Information

**Additional file 1: Figure S1.** Frequency distributions of traits measured in the WW (blue), Dr1 (red) and Dr2 (black) trials, in the RIL population. Mean parental trait values are indicated with a dashed line.

**Additional file 2: Table S1.** Custom database for candidate gene mining, with peptide sequences retrieved from Ensembl Plants.

**Additional file 3: Table S2. **QTL selected for candidate gene mining.

**Additional file 4: Table S3.** BLASTp results.

**Additional file 5: Table S4.** GO term enrichment.

**Additional file 6: Table S5.** Experimental summary of the three greenhouse trials which were used to grow the recombinant inbred line mapping population.

**Additional file 7: Table S6.** Average phenotype data for the *L. sativa* cv. Salinas x *L. serriola* RIL population (genotypes 1-371).

## Data Availability

The phenotypes generated and analysed in thus study, specified by genotype are presented in Supplementary file Table S6, whilst the genomic data are available in the public domain from the lettuce genome (V8, genome ID: 467) in the Phytozyme v13 database (https://phytozome-next.jgi.doe.gov/info/Lsativa_V8). All other data are available from the corresponding author on request.

## Abbreviations

WUE: Water-use efficiency
QTL: Quantitative trait loci
LOD: Logarithm of odds
LG: Linkage group
PV: Phenotypic variation
g_s_: Stomatal conductance
E: Transpiration
IR: Infra-red thermal imaging
Δ^13^: Carbon isotope discrimination
