# Supplementary figures and images for: The genetic basis of water‐use efficiency and yield in lettuce
Source: BMC Plant Biol. 2021 May 27;21:237. doi: 10.1186/s12870-021-02987-7 (PMC8157645; doi:10.1186/s12870-021-02987-7)

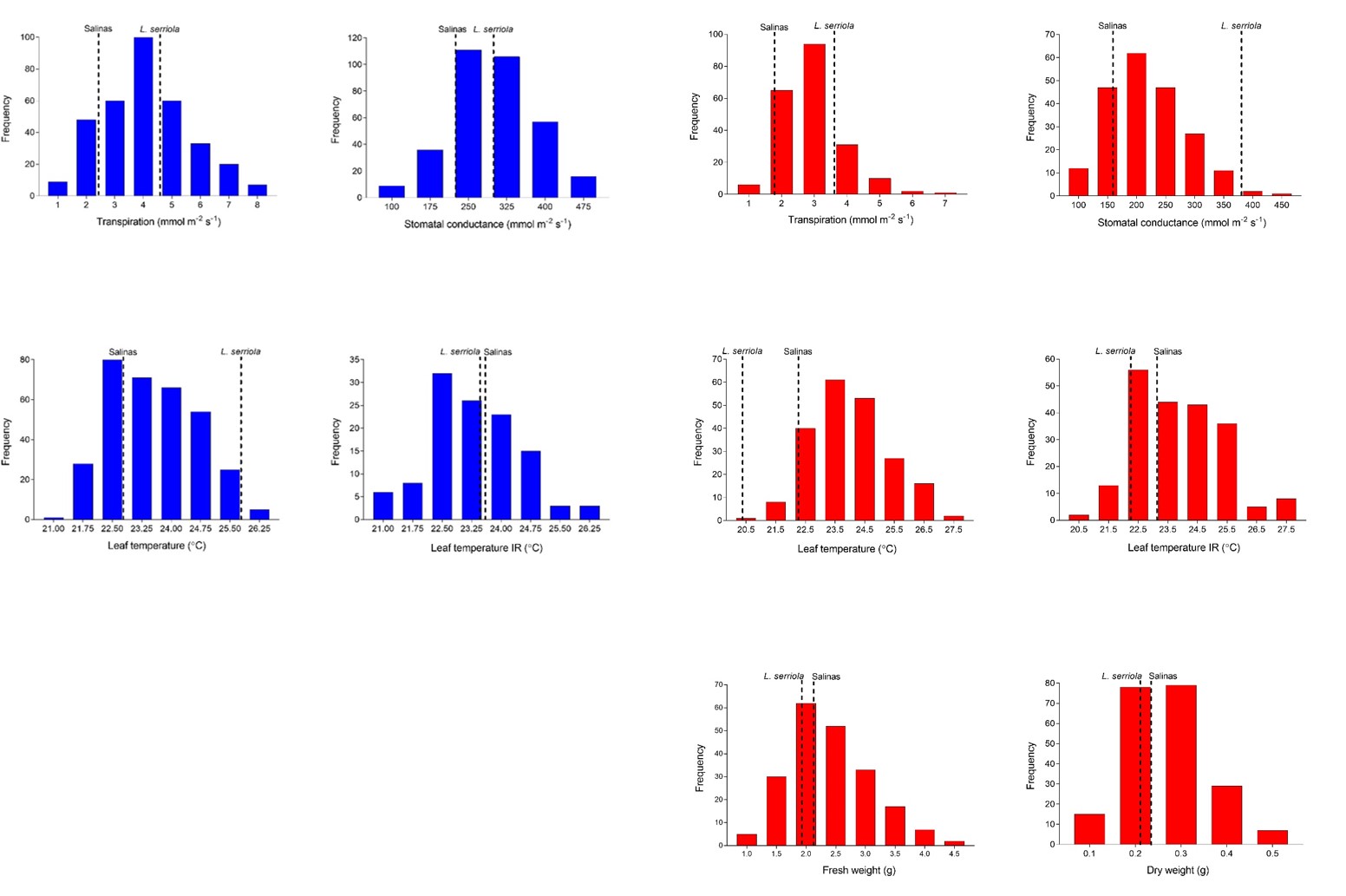

Supplement: Supplementary file 1 — Additional file 1: Figure S1. Frequency distributions of traits measured in the WW (blue), Dr1 (red) and Dr2 (black) trials, in the RIL population. Mean parental trait values are indicated with a dashed line. [file 12870_2021_2987_MOESM1_ESM.zip › FigureS1a.jpg]

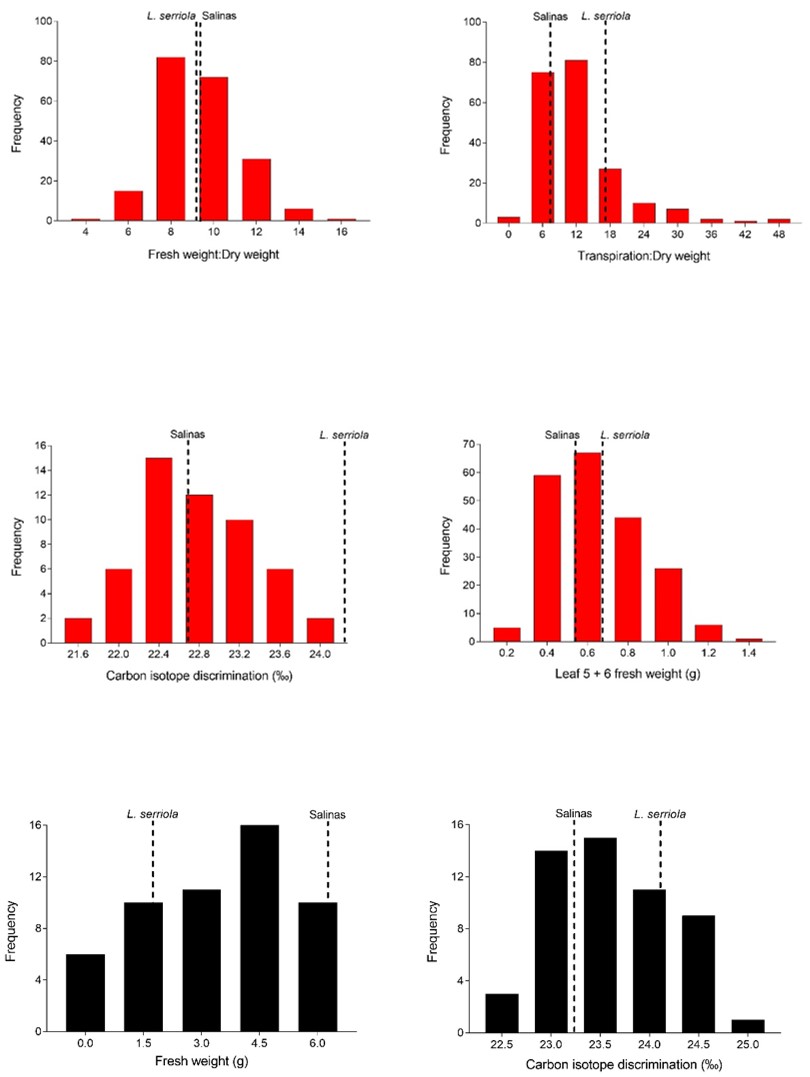

Supplement: Supplementary file 1 — Additional file 1: Figure S1. Frequency distributions of traits measured in the WW (blue), Dr1 (red) and Dr2 (black) trials, in the RIL population. Mean parental trait values are indicated with a dashed line. [file 12870_2021_2987_MOESM1_ESM.zip › FigureS1b.jpg]
